# Molecular fingerprinting reflects different histotypes and brain region in low grade gliomas

**DOI:** 10.1186/1471-2407-13-387

**Published:** 2013-08-15

**Authors:** Samantha Mascelli, Annalisa Barla, Alessandro Raso, Sofia Mosci, Paolo Nozza, Roberto Biassoni, Giovanni Morana, Martin Huber, Cristian Mircean, Daniel Fasulo, Karin Noy, Gayle Wittemberg, Sara Pignatelli, Gianluca Piatelli, Armando Cama, Maria Luisa Garré, Valeria Capra, Alessandro Verri

**Affiliations:** 1 Neurosurgery Unit, Istituto Giannina Gaslini, via G. Gaslini 5, 16147, Genoa, Italy; 2 DISI - Department of Computer Science, Università degli Studi di Genova, Via Dodecaneso, 35-16146, Genoa, Italy; 3 Pathology Unit, Istituto Giannina Gaslini, via G. Gaslini 5, 16147, Genoa, Italy; 4 Molecular Medicine Unit, Istituto Giannina Gaslini, via G. Gaslini 5, 16147, Genoa, Italy; 5 Neuroradiology Unit, Istituto Giannina Gaslini, via G. Gaslini 5, 16147, Genoa, Italy; 6 Siemens AG, Corporate Technology, Freyeslebenstr. 1, 91058, Erlangen, Germany; 7 SCR - Siemens Corporate Research, Princeton, NJ, USA; 8 Neuro-oncology Unit, Istituto Giannina Gaslini, via G. Gaslini 5, 16147, Genoa, Italy

**Keywords:** Gene expression profile, Machine learning, Low-grade glioma, PA, Mixed glial-neuronal tumours

## Abstract

**Background:**

Paediatric low-grade gliomas (LGGs) encompass a heterogeneous set of tumours of different histologies, site of lesion, age and gender distribution, growth potential, morphological features, tendency to progression and clinical course. Among LGGs, Pilocytic astrocytomas (PAs) are the most common central nervous system (CNS) tumours in children. They are typically well-circumscribed, classified as grade I by the World Health Organization (WHO), but recurrence or progressive disease occurs in about 10-20% of cases. Despite radiological and neuropathological features deemed as classic are acknowledged, PA may present a bewildering variety of microscopic features. Indeed, tumours containing both neoplastic ganglion and astrocytic cells occur at a lower frequency.

**Methods:**

Gene expression profiling on 40 primary LGGs including PAs and mixed glial-neuronal tumours comprising gangliogliomas (GG) and desmoplastic infantile gangliogliomas (DIG) using Affymetrix array platform was performed. A biologically validated machine learning workflow for the identification of microarray-based gene signatures was devised. The method is based on a sparsity inducing regularization algorithm *l*_*1*_*l*_*2*_ that selects relevant variables and takes into account their correlation. The most significant genetic signatures emerging from gene-chip analysis were confirmed and validated by qPCR.

**Results:**

We identified an expression signature composed by a biologically validated list of 15 genes, able to distinguish infratentorial from supratentorial LGGs. In addition, a specific molecular fingerprinting distinguishes the supratentorial PAs from those originating in the posterior fossa. Lastly, within supratentorial tumours, we also identified a gene expression pattern composed by neurogenesis, cell motility and cell growth genes which dichotomize mixed glial-neuronal tumours *versus* PAs. Our results reinforce previous observations about aberrant activation of the mitogen-activated protein kinase (MAPK) pathway in LGGs, but still point to an active involvement of TGF-beta signaling pathway in the PA development and pick out some hitherto unreported genes worthy of further investigation for the mixed glial-neuronal tumours.

**Conclusions:**

The identification of a brain region-specific gene signature suggests that LGGs, with similar pathological features but located at different sites, may be distinguishable on the basis of cancer genetics. Molecular fingerprinting seems to be able to better sub-classify such morphologically heterogeneous tumours and it is remarkable that mixed glial-neuronal tumours are strikingly separated from PAs.

## Background

Primary intra-axial paediatric low grade tumours include pilocytic astrocytoma (PA), pilomyxoid astrocytoma, diffuse fibrillary astrocytoma (FA), ganglioglioma (GG), desmoplastic infantile ganglioglioma (DIG), desmoplastic infantile astrocytoma (DIA) and dysembryoplastic neuroepithelial tumour [1]. For brevity, they will be thereafter defined LGG.

PA is the most common central nervous system (CNS) tumour, representing approximately 21-23% of all primary brain tumours in children [2]. It is typically a well-circumscribed, contrast-enhancing astrocytic neoplasm with prolonged overall survival and high complete remission rates [3]. PA arises most commonly in the cerebellum, but can be found anywhere, including the cerebral hemispheres, thalamus and hypothalamus, brainstem, optic pathways, and spinal cord [3,4].

Reflecting the generally slow growth and low proliferative potential of LGGs, complete surgical resection is the preferred therapeutic choice. Unfortunately, gross total resection is not attainable in many of these tumours that are centrally located which, in about 10 to 20% of the cases, despite adjuvant treatment, tend to recur or show progressive growth [5,6]. Most importantly, rare examples of PA undergo malignant transformation, even if completely resected [7,8].

Despite radiological and neuropathological features deemed as classic are acknowledged, PA may present a bewildering variety of microscopic features, including a wide range of tissue patterns, most of which may be found within the same lesion. It is worth remembering that both normal and neoplastic astrocytes exhibit molecular and functional heterogeneity [9-13].

The tumours containing both neoplastic ganglion and astrocytic cells are rare, representing less than an hundredth of the tumours of CNS and its coverings. Such tumours, which belong to the neuronal and mixed glial-neuronal tumours of the WHO classification and corresponding to grade I, comprise gangliogliomas (GG), and gangliogliomas with desmoplasia, i.e. desmoplastic infantile gangliogliomas (DIG) both typically arising from the telencephalon [2]. The differential diagnosis may be difficult due to small biopsy size. Moreover, the glial component of a ganglioglioma may be pilocytic looking (this is as much as to say that a pilocytic morphology is considered completely acceptable in a ganglioglioma). Lack of specific immunohistochemical, cytogenetic, or molecular markers increases difficulties in classification.

The expanding utilization of high-throughput technologies to study paediatric brain tumours will likely change how they are both classified and treated henceforward [5,6]. In this field, the use of microarrays has been expanding exponentially to several areas such as genetic screening, safety assessment and diagnostics [14], but repeatability of published microarray studies is apparently limited [15,16]. In the neuro-oncological context, a LGG genotype-phenotype correlation still remains an open problem [17,18]. Gene signatures able to classify LGGs in accordance with clinical and biological features were provided [9,19-22]. Nevertheless, a complete genetic landscape of paediatric PA is still missing and the specific molecular signatures able to correlate their phenotype (brain sites and heterogeneous histotypes) to their genotype still remain to be studied in depth.

Keeping this in mind, we aimed to identify a molecular fingerprinting able to reflect different histotypes and brain region in LGGs. In particular, the study addressed three different biological questions: (1) characterize supratentorial vs. infratentorial LGGs, (2) identify a specific characterization for the PAs based upon site of lesion, and (3) discriminate, within supratentorial neoplasms, mixed glial-neuronal tumours vs. PAs.

This relatively simple, albeit fraught with meaning, goal gave us the opportunity to develop a robust and validated experimental workflow, paving the way for future studies, whose goal will be the identification of gene fingerprints explicitly correlated to clinical parameters.

## Methods

**Figure 1:**
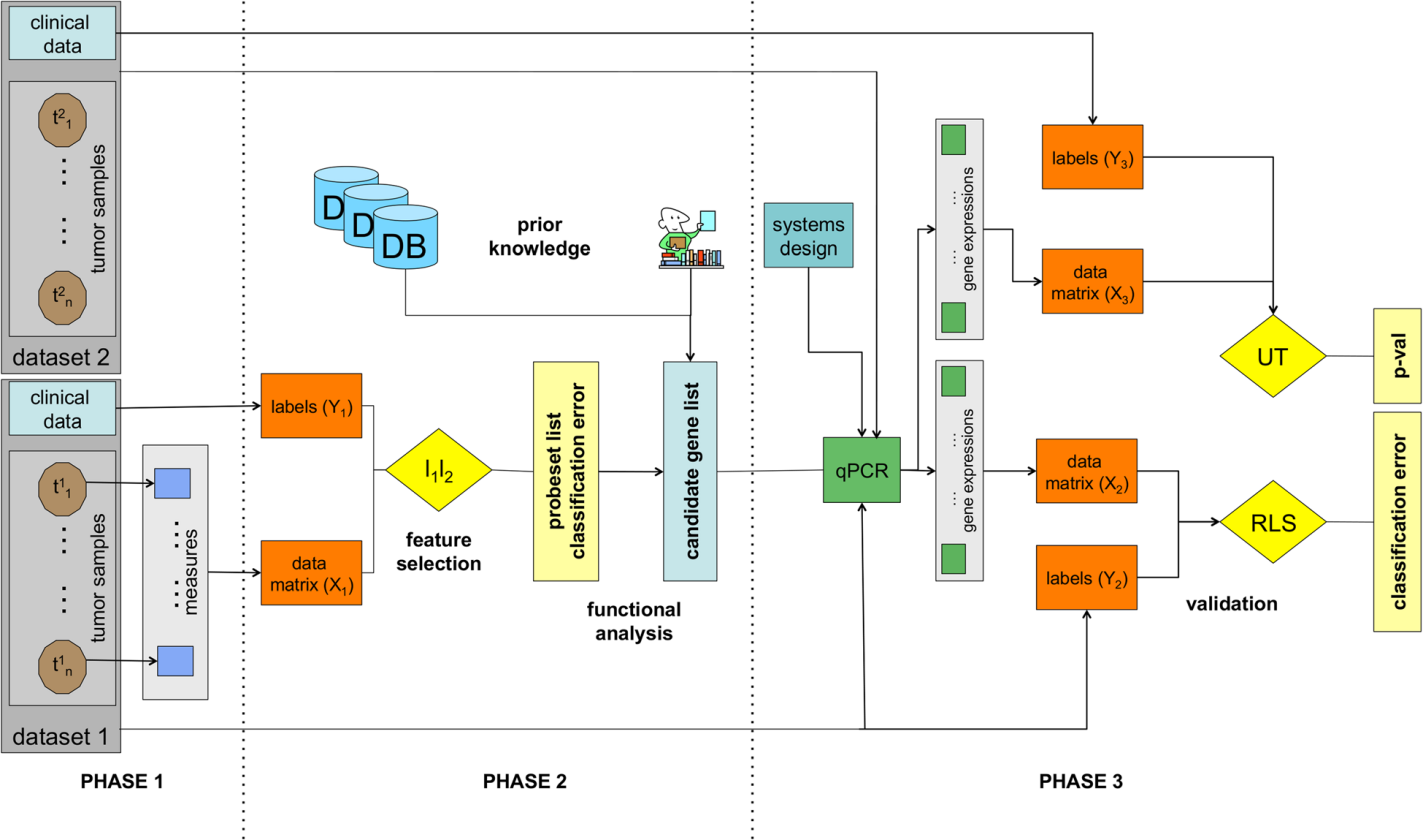
**Workflow.** This figure depicts the entire workflow consisting in several computational and biological procedures. The three main phases are indicated as Phase 1 (data preparation), Phase 2 (statistical analysis and candidate gene list identification) and Phase 3 (validation).

We adopted a biologically validated method to identify reliable and predictive gene expression signatures on tumour data. The pipeline, represented in Figure 1, is a supervised machine learning workflow consisting in 3 main consecutive phases: case selection and tumour specimen processing, unbiased *l*_*1*_*l*_*2*_ feature selection framework with functional characterization of the gene signature, and real-time quantitative reverse transcription-PCR (qPCR). Detailed description of the pipeline is reported in Additional file 1.

### Case selection and tumour processing

A series (dataset 1) of 40 paediatric primary LGGs who underwent surgery from 1991 to 2009 at the Neurosurgery Unit of the Giannina Gaslini Children’s Hospital were selected and enrolled in the study. The inclusion criteria were diagnosis of PA or ganglioglioma with or without desmoplasia, i.e. GG or DIG; the availability of complete clinical data and fresh frozen tissue specimen with a tumour cell content of at least 80%, while exclusion criteria were lack of histological diagnosis and the presence of extensive dissemination. The cohort included 27 PAs, 12 mixed glial-neuronal tumours (7 GGs and 5 DIGs) and one FA. Seventeen tumours arose in infratentorial regions, while 23 were supratentorial. The FA was forced in the first phase of analysis and partially during the second phase in order to have an internal control. Only one case was associated with a genetic syndrome, namely Neurofibromatosis type 1 (NF1) [MIM ID #162200]. The male/female ratio of 1.2:1, and the mean age 7 years (range 5 months to 17 years). The main clinical-pathological features are summarized in Table 1. The sections were reviewed by the local neuropathologist (P.N.) and the tumours were classified according to the WHO classification [3]. The sets of samples are formed to precisely answer the biological questions of interest. Moreover, the sets were made the more homogeneous possible in order to minimize the undesiderable effects of the inter-tumoural genetic differences due to the intrinsic constitutional variations among individuals.

**Table 1:** Clinical data

***ID***	***Age***	***Gender***	***Diagnosis***	***WHO***	***Site***
	***(months)***			***grade***	
1	35	F	PA	I	Infratentorial
2	30	M	PA	I	Infratentorial
3	29	M	PA	I	Infratentorial
4	38	F	PA	I	Infratentorial
5	37	M	PA	I	Infratentorial
6	42	F	PA	I	Infratentorial
7	16	F	PA	I	Infratentorial
8	46	F	PA	I	Infratentorial
9	41	M	PA	I	Infratentorial
10	33	M	PA	I	Infratentorial
11	143	F	PA	I	Infratentorial
12	82	F	PA	I	Infratentorial
13	165	F	PA	I	Infratentorial
14	48	F	PA	I	Infratentorial
15	67	M	PA	I	Infratentorial
16	149	M	PA	I	Infratentorial
17	36	F	PA	I	Infratentorial
18	161	M	PA	I	Supratentorial
19	104	F	PA	I	Supratentorial
20	176	M	PA	I	Supratentorial
21	153	M	PA	I	Supratentorial
22	108	F	PA	I	Supratentorial
23	188	M	PA	I	Supratentorial
**24**	**128**	**M**	**PA**	**I**	**Supratentorial**
25	32	F	PA	I	Supratentorial
26	5	F	PA	I	Supratentorial
27	108	M	PA	I	Supratentorial
28	171	M	FA	II	Supratentorial
29	11	F	DIG	I	Supratentorial
30	27	F	DIG	I	Supratentorial
31	6	F	DIG	I	Supratentorial
32	6	M	DIG	I	Supratentorial
33	13	M	DIG	I	Supratentorial
34	14	F	GG	I	Supratentorial
35	155	M	GG	I	Supratentorial
36	119	F	GG	I	Supratentorial
37	189	F	GG	I	Supratentorial
38	149	M	GG	I	Supratentorial
39	199	F	GG	I	Supratentorial
40	130	F	GG	I	Supratentorial

For each patient we report the corresponding ID, age at surgery (in months), gender (F = female, M= male), diagnosis (PA = pilocytic astrocytoma, FA = diffuse fibrillary astrocytoma, DIG = desmoplastic infantile ganglioglioma, GG = ganglioglioma and site of lesion. Patient ID24 reported in bold is associated to the genetic syndrome NF1.

Total RNA was extracted from serial frozen sections of tumour tissue by using the TRIzol reagent combined with silica column purification system (Invitrogen, Carlsbad, CA). Quantification and quality assurance were performed using the NanoDrop spectrophotometer (NanoDrop Technologies Wilmington, Delawere USA) and the Agilent 2100 bioanalyzer (Agilent Technologies, Waldbronn, Germany), respectively. Double-stranded cDNA were processed according to the Affymetrix GeneChip Expression Analysis Technical Manual (Affymetrix, Santa Clara, CA). Microarray data for 40 LGG samples was generated with Affymetrix HG-U133Plus2.0 arrays (Affymetrix, Santa Clara, CA). Gene expressions were extracted from the .CEL files and normalized using the Robust Multichip Average method [23] by running an R [24] script, based on the aroma package [25]. The dataset for the microarray experiment was uploaded in the Gene Expression Omnibus public repository at National Center for Biotechnology Information [accession number GSE28238].

Written informed consent was obtained from all the patients’parents or guardians and the local Ethics Committee for human studies approved the research.

### Unbiased l_1_l_2_ feature selection framework

The feature selection method we adopted is a regularization method capable of selecting subsets of discriminative genes, namely l_1_l_2_ regularization with double optimization. The algorithm can be tuned to give a minimal set of discriminative genes or larger sets including correlated genes. The method is based on the optimization principle presented in [26] and further developed and studied in [27,28].

The l_1_l_2_ with double optimization algorithm looks for a linear function (model), whose sign gives the classification rule that can be used to associate a new sample to one of the two classes. The output function is a sparse model, i.e. some input variables (probe-sets) will not contribute to the final estimator. The algorithm is based on the minimization of a functional depending on a least square error term combined with two penalties. The least square term ensures fitting of the data whereas adding the two penalties allows to avoid over-fitting. The role of the two penalties is different, the l_1_ term (sum of absolute values) enforces the solution to be sparse, the l_2_ term (sum of the squares) preserves correlation among the variables. The training for selection and classification requires the choice of the regularization parameters for both l_1_l_2_ regularization and regularized least squares (RLS) denoted with τ* and λ*, respectively. In fact model selection and statistical significance is performed within two nested K-cross validation loops as in [29,30]. Being interested in a comprehensive list of relevant variables we fixed our attention on the lists obtained with the highest values for the correlation parameter μ.

The statistical framework described above provides a set of K lists of selected variables, therefore it is necessary to choose an appropriate criterion [31] in order to assess a common list of relevant variables (probe-sets or proteins, in our case). We based ours on the absolute frequency, i.e. we decided to promote as relevant variables the most stable probe-sets across the lists. The threshold we used to select the final lists was chosen according to the slope variation of the number of selected genes vs. frequency (plot not shown), its value being 70%. In this way we manage to cut out those variables that are not stable across the cross-validation lists, similarly to the procedure adopted in [30]. We also visualized the signatures in heat-map plots and 3d visualizations of classified samples.

### Functional characterization of the gene signature

Multiple probe-sets for a gene were collapsed to one entry per gene, based on the best frequency score. Non-mapping or non-coding probe-sets were discarded. The National Institute of Health Database for Annotation, Visualization and Integrated Discovery (DAVID) web-tool [32] was used to identify structural, functional, and pathway categories in the selected list. The analysis also ranked in detail the Gene Ontology (GO) [33] terms in the Biological Process (BP) domain including the identified probe-sets. The functional annotation was performed using the Expression Analysis Systematic Explorer (EASE) [34] with structural and functional class data from the GO, GenBank and UniGene databases, and with pathway data from Gene Map Annotator and Pathway Profiler (GenMAPP) [35], the Kyoto Encyclopedia of Genes and Genomes (KEGG) [36] and the Biocarta [37] databases. The Exploratory Gene Association Networks (EGAN) Java desktop application was also used to visualize the interactions among the selected genes [38].

### Real-time quantitative reverse transcription-PCR

Following the same criteria for the case selection, we chose an additional set of patients (dataset 2), composed by 14 PAs and 4 mixed glial-neuronal tumours, in order to confirm and validate with qPCR the most significant genetic signatures emerging from gene-chip analysis. Each systems were in-house designed by a fine tuning procedure as described [39]. Specific primers were developed targeting: *ABBA1* [NM 138383], *APOD* [NM 001647], *ARX* [NM 139058], *CXCL14* [NM 004887], *FOSB* [NM 001114171], *FOXG1* [NM 005249], *GPR17* [NM 001161415], *LHX2* [NM 004789], *NRXN2* [NM 015080], *PTGD2S* [NM 000954], *SDC3* [NM 014654], *SNX22* [NM 024798], *SPOCK1* [NM 004598], *TIMP4* [NM 003256] and *ZFHX4* [NM 024721]. Primers sequences and the amplification conditions are reported in Additional file 2. Beta actin (*ACTB*) [NM 001101], Pyruvate kinase (*PKM2*) [NM 002654] and Beta-2-microglobulin (*B2M*) [NM 004048] were used as the endogenous control genes for each tumour specimen [39]. Amplifications were performed using an ABI PRISM 7500 HT Sequence Detection System (Applied Biosystems, Foster City, CA) and primer concentrations were adjusted accordingly to the assays temperature. Validation of each system was performed using standard curves (SCs) on cDNA derived from the 1603-MED medulloblastoma cell line [40].

The reproducibility of the calibration curve was analyzed qPCR efficiencies of each system were calculated as described [41]. The relative quantification of genes transcript was performed according to the comparative method (2^-ΔCt^), Applied Biosystems User Bulletin no. 2P/N 4303859) [42,43], using the value emerged by geometric mean of *B2M*, *PKM2* and *ACTB* as the normalizer (Ct_*ref*_). Gene expression levels of the 18 candidates were calculated for each LGG sample by the 2^-ΔΔCt^ equation using as ΔCt_*ref*_ the median ΔCt value among all cases. The Minimum Information for Publication of qPCR Experiments (MIQE) are provided [44].

### Statistical validation

Comparisons of the quantitative data of gene expressions were performed by the Mann–Whitney U test since the normality and homoscedasticity assumptions were not fulfilled. Statistical tests were 2-sided, and a p-value less than 0.05 was considered statistically significant.

We also performed a multivariate data analysis by employing the algorithm known as Regularized Least Squares (RLS) [45]. The algorithm is based on the minimization of a functional depending on a least square error term combined with a regularization term, *i.e.,* the l_2_ term (sum of the squares). Similarly to the l_1_l_2_ algorithm, RLS is run in a double nested cross-validation framework to avoid selection bias.

## Results

### Biologically validated molecular fingerprint of infratentorial *versus* supratentorial LGGs

We conducted a high-resolution analysis of genome-wide expression patterns on 40 paediatric LGGs, including 17 arising in infratentorial and 23 in supratentorial regions (Table 1), using Affymetrix HG-U133 Plus 2.0 chip-arrays. To select a list of highly discriminative probe-sets, we applied the l_1_l_2_ selection statistical framework to the dataset. The system performance was evaluated by its corresponding cross-validation error, as low as 8%. The resulting list, reported in Additional file 3, consists of 331 probe-sets, sorted according to their frequency score and corresponding to the maximum value of the correlation parameter μ.

**Figure 2:**
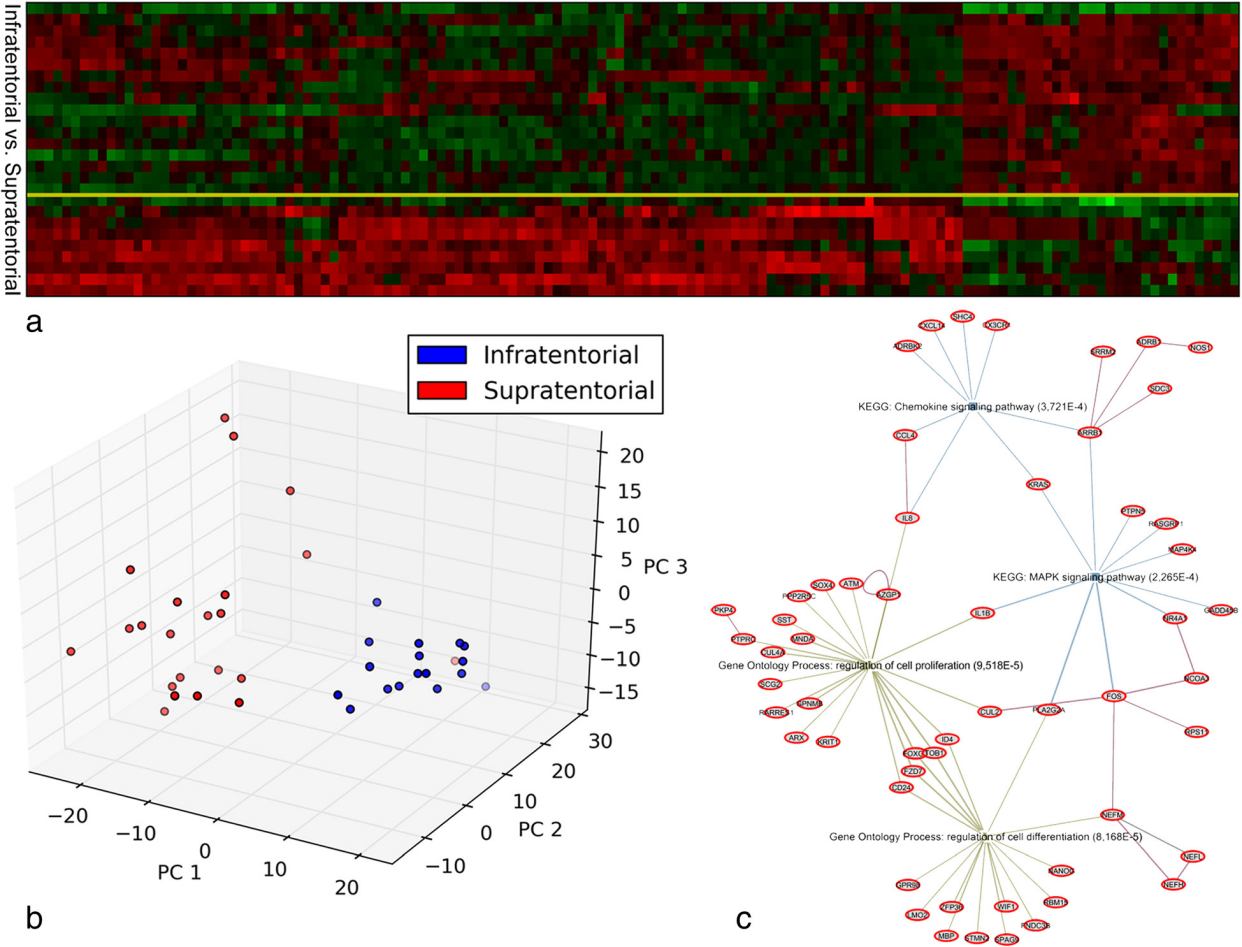
**Infratentorial vs. Supratentorial LGGs. a)** Heatmap - infratentorial *vs.* supratentorial LGGs. Heatmap plot of the gene expression submatrix of the 40 samples restricted to the 331 probe-sets selected by the l_1_l_2_ feature selection. The tumours are grouped according to the lesion site (infratentorial *vs*. supratentorial). Each column represents a sample and each row is associated to a probe-set. The relative expression of the probe-sets is normalized ranging from 0 (blue, under-represented) to 1 (red, over-expressed); **b)**. This figure illustrates a 3-dimensional visualization of the dataset restricted to the 331 selected probe-sets. The 3D representation is obtained by projecting the data submatrix onto its 3 principal components *i.e.* the components of maximum variance. Red circles represent the supratentorial and the blue circles the infratentorial LGGs; **c)**. EGAN sotware provide a hypergraph visualization showing how enriched gene sets/pathways/GO connect significant genes from LGGs signature along with standard protein-protein interaction.

The strong discriminative power of the selected probe-sets is depicted by either a heat-map plot or a multivariate representation (Figure 2a,b). The FA case was not classified neither supratentorial nor infratentorial by the l_1_l_2_ algorithm, showing the robustness of the method.

In order to obtain a 3D visualization, the expression data restricted to the 331 probe-sets was projected on its first 3 principal components, *i.e*., the components of maximum variance. It is evident that the two classes are clearly separated in the multidimensional space (Figure 2b). In Table 2 we list the selected genes and the highest frequency score associated to each of them.

**Table 2:** **Infratentorial vs Supratentorial LGGs: selected genes by** l_1_l_2_

***Gene***	***f.s.***	***Gene***	***f.s.***	***Gene***	***f.s.***	***Gene***	***f.s.***	***Gene***	***f.s.***
ABBA1	100	KIAA2022	100	KIAA1189	98	RHOB	92	ZNF228	78
ADRBK2	100	NRGN	100	KLHDC8A	98	RORB	92	LTB4DH	75
ALK	100	**PAX3**	**100**	LRAP	98	SCG2	92	TMEPAI	75
AMMECR1	100	PCDH8	100	MAN2A1	98	SLC10A4	92	BMS1P5	72
ANKRD10	100	PCDHB16	100	NEFH	98	SNRPN	92	KIAA1967	72
**APOD**	**100**	PCNX	100	NHSL1	98	SOX4	92	KRAS	72
ARRB1	100	PDGFRA	100	NOS1	98	SPRN	92	MNDA	72
ARX	100	PKIB	100	NR4A1	98	TOB1	92	CUL4A	70
ATP6V0E1	100	PPM1E	100	PLK2	98	AKAP11	90	NRXN2	70
ATXN3	100	PPP1R9A	100	POLR2J2	98	FCF1	90	VSNL1	70
BICD1	100	PRO1073	100	POSTN	98	GFAP	90		
RBM15	100	RARRES1	100	PSD3	98	GPR98	90		
C1QL1	100	RASGRP1	100	RPS11	98	MAP4K4	90		
CAPN3	100	RCOR1	100	SPAG9	98	RAB27B	90		
CCDC76	100	RGS4	100	SPATA18	98	SHC4	90		
CD24	100	RRM2	100	SPOCK1	98	VAMP1	90		
**CHAD**	**100**	SDC3	100	STON2	98	CRYAB	88		
CNIH3	100	SHROOM3	100	TMEM158	98	DOCK9	88		
COCH	100	SLC35A3	100	TSPAN5	98	FAM149A	88		
COG5	100	SMA4	100	ZC3H7A	98	FNDC3B	88		
CREB5	100	SMOC1	100	**ADARB1**	**95**	FOSB	88		
CTGLF1	100	SNX21	100	CAMK2N1	95	N4BP2	88		
CX3CR1	100	SOX10	100	CAMKK2	95	NUCKS1	88		
CXCL14	100	STMN2	100	CDC2L5	95	RPL37A	88		
CYR61	100	STXBP6	100	COL22A1	95	SST	88		
ENC1	100	SUSD5	100	GLTSCR2	95	ZNF226	88		
EPHX1	100	**TIMP4**	**100**	GPNMB	95	GRM3	85		
F2RL1	100	TMTC4	100	GRIA4	95	KIAA1919	85		
FBXL3	100	TNFAIP6	100	LRRFIP1	95	PGM2L1	85		
FNDC1	100	TRPM3	100	LYZ	95	**PTPRC**	**85**		
FOS	100	U2AF1	100	NANOG	95	CCL4	82		
**FOXG1**	**100**	**ZFHX4**	**100**	OLFM2	95	CUL2	82		
FZD7	100	ZFP36	100	OPA1	95	FBXL11	82		
GADD45B	100	ZNF207	100	PHCA	95	GARNL4	82		
GNL3L	100	ZNF294	100	PKP4	95	LCMT2	82		
GUCY1A3	100	ADRB1	98	PLEKHA2	95	TMEM132E	82		
HINT3	100	ALDOC	98	PMP2	95	TYMS	82		
HS3ST3B1	100	ANKRD22	98	PPP2R5C	95	GGNBP2	80		
ID4	100	ATM	98	WIF1	95	**IL8**	**80**		
**IRX2**	**100**	CCDC91	98	ASCL1	92	PLA2G2A	80		
KIAA0101	100	CLEC4A	98	AZGP1	92	PMS2L5	80		
KRIT1	100	COL9A2	98	F11R	92	S100A1	80		
**LHX2**	**100**	ENPP2	98	FZD8	92	SEZ6L	80		
**MBP**	**100**	FAM107B	98	LCAT	92	ZNF423	80		
MICAL2	100	FAM89A	98	LMO2	92	EPHA5	78		
NAIP	100	FRMD4A	98	MIAT	92	GPBP1L1	78		
NCOA3	100	GALNT13	98	NEFM	92	MCTP1	78		
NEFL	100	**GAS7**	**98**	PPP1CB	92	PTPN5	78		
**NR2E1**	**100**	GPR17	98	PTGD2S	92	STMN4	78		

List of 206 gene symbols selected by the l_1_l_2_ procedure. For each Gene ID we report the highest frequency score. We share 14 genes, showed in bold, with the results reported by previous studies [9,19,20].

Over-representation analysis using DAVID web-tool revealed that the main GO terms in the biological process (BP) domain include: neuronal development, brain morphogenesis and anatomical structure development. Thanks to the EGAN software program, that interfaces with existing GO and literature annotation of the genes and with canonical pathways to perform enrichment statistics, molecular networks based on direct or indirect gene-gene interactions were created for the list of 206 genes we identified. The most enriched pathways are chemokine signaling, mitogen-activated protein kinase (MAPK) signaling, T cell receptor signaling and cell adhesion molecules (CAMs) pathways (Figure 2c).

Using the available WEB-based gene set analysis tools, a functionally based criterion was then applied to the list of 206 genes in order to select groups of genes that were most represented in the tumour development pathways and that were top-ranked in the l_1_l_2_ list. The resultant minimal list was composed by 19 out of 331 probe-sets, corresponding to 15 loci, see Table 3. Relative functional analysis showed that the selected genes enriched BP related to CNS neuron differentiation, forebrain development, regulation of metabolic process, and cell proliferation. A brief comment of each locus is reported in Additional file 4, listing the main protein functions for the 15 genes that significantly discriminate infratentorial *versus* supratentorial LGGs.

**Table 3:** Molecular fingerprint of LGGs composed by the selected 15 genes

**Gene/Locus**	**Description**	**Biological Process**
FOXG1 / 14q13	forkhead box G1	GO:0007417 CNS development GO:0022008 neurogenesis
		GO:0030900 forebrain development GO:0048699 generation of neurons
		GO:0021954 CNS neuron development GO:0021953 CNS neuron differentiation
		GO:0048666 neuron development GO:0007423 sensory organ development
		GO:0043583 ear development GO:0009953 dorsal/ventral pattern formation
		GO:0006355 regulation of transcription, DNA-dependent
		GO:0051252 regulation of RNA metabolic process
		GO:0045449 regulation of transcription
		GO:0019219 regulation of nucleobase, nucleoside, nucleotide and nucleic acid
		GO:0048667 cell morphogenesis involved in neuron differentiation
		GO:0048812 neuron projection morphogenesis
		GO:0048858 cell projection morphogenesis GO:0032990 cell part morphogenesis
		GO:0031175 neuron projection development
GPR17 / 2q21	G protein-coupled receptor 17	GO:0007165 signal trasduction
		GO:0007186 G-protein coupled receptor protein signaling pathway
CXCL14 / 5q31	chemokine (C-X-C motif)	GO:0006995 immune response GO: 0006935 chemotaxis
	ligand 14	GO:0007267 cell-cell signaling GO:0007165 signal trasduction
ARX / Xp21	aristaless related homeobox	GO:0007417 CNS development, GO:0022008 neurogenesis
		GO:0030900 forebrain development, GO:0048699 generation of neurons
		GO:0021954 CNS neuron development, GO:0021987 cerebral cortex development
		GO:0021543 pallium development
		GO:0006355 regulation of transcription, DNA-dependent
		GO:0051252 regulation of RNA metabolic process
		GO:0045449 regulation of transcription
		GO:0019219 regulation of nucleobase, nucleoside, nucleotide and nucleic acid
		GO:0048667 cell morphogenesis involved in neuron differentiation
		GO:0048812 neuron projection morphogenesis
		GO:0048858 cell projection morphogenesis
		GO:0032990 cell part morphogenesis
		GO:0031175 neuron projection development
LHX2 / 9q33q34.1	LIM homeobox 2	GO:0007417 CNS development GO:0022008 neurogenesis
		GO:0030900 forebrain development GO:0048699 generation of neurons
		GO:0048666 neuron development GO:0009953 dorsal/ventral pattern formation
		GO:0006355 regulation of transcription, DNA-dependent
		GO:0051252 regulation of RNA metabolic process
		GO:0045449 regulation of transcription
		GO:0019219 regulation of nucleobase, nucleoside, nucleotide and nucleic acid
		GO:0048667 cell morphogenesis involved in neuron differentiation
		GO:0048812 neuron projection morphogenesis
		GO:0048858 cell projection morphogenesis
		GO:0032990 cell part morphogenesis, GO:0031175 neuron projection development
TIMP4 / 3p25	TIMP metallopeptidase	GO:0007417 CNS development GO:0009725 response to hormone stimulus
	inhibitor 4	GO:0032496 response to lipopolysaccharide
		GO:0034097 response to cytokine stimulus
APOD / 3q26.2qter	apolipoprotein D	GO.0006629 lipid metabolic process
PTGD2S / 9q34.2-34.3	prostaglandin D2 synthase	GO:0006633 fatty acid biosynthetic process
	21 kDa (brain)	GO:0006810 transport
SDC3 / 1pter-p22.3	syndecan 3	GO:0007155 cell adhesion
NRXN2 / 11q13	neurexin 2	GO:0007268 synaptic trasmission GO:0007269 neurotransmitter secretion
		GO:0007416 synapse assembly GO:0007155 cell adhesion
SNX22 / 15q22.31	sorting nexin 22	GO:0007165 signal trasduction GO:0007154 cell comunication
ZFHX4 / 8q21.11	zinc finger homeobox 4	GO:0006355 regulation of transcription, DNA-dependent
		GO:0051252 regulation of RNA metabolic process
		GO:0045449 regulation of transcription
		GO:0019219 regulation of nucleobase, nucleoside, nucleotide and nucleic acid
		GO:0015031 protein transport
SPOCK1 / 5q31	testican 1	GO:0007417 CNS development GO:0007165 signal trasduction
		GO:0007155 cell adhesion
ABBA1 / 16q22.1	metastasis suppressor 1 like	GO:0007165 signal trasduction
FOSB / 19q13.32	FBJ murine osteosarcoma	GO:0006355 regulation of transcription, DNA-dependent
	viral oncogene homolog B	GO:0051252 regulation of RNA metabolic process
		GO:0045449 regulation of transcription
		GO:0019219 regulation of nucleobase, nucleoside, nucleotide and nucleic acid

Gene annotations and GO Biological Process terms for the minimal list of 15 genes, selected for the biologically validated molecular fingerprint of LGGs related to site of lesion (infratentorial *vs* supratentorial).

### qPCR analysis

**Figure 3:**
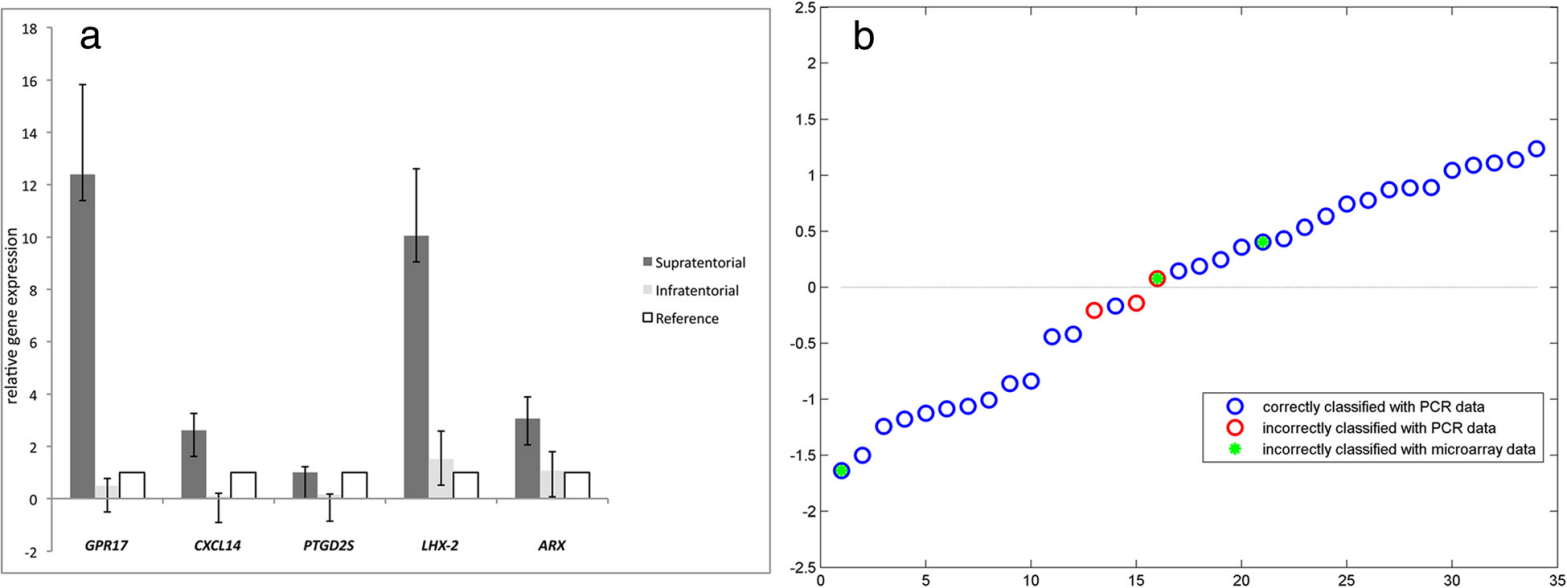
**The best differentially expressed genes with qPCR between infratentorial and supratentorial LGGs. a)** the best differentially expressed genes with qPCR between infratentorial and supratentorial LGGs. Relative gene expression for the best 5 differentially expressed genes selected with univariate Mann–Whitney test; **b)** in this plot we show the comparison between the microarray and the values of the estimated RLS classification function for each sample measured by both qPCR and microarray. The negative values are assigned to the infratentorial site, while positives are the supratentorial ones. The blue circles correspond to the correctly classified cases by qPCR. The red circles indicate the misclassified samples by qPCR. The green dots are the misclassified samples by the microarray model.

In order to confirm and validate the results of microarray analyses, we considered 52 samples measured with qPCR, whose 34 samples from dataset 1 subjected to microarray experiments and 18 samples from dataset 2, on which only qPCR was performed (see Figure 1). The relative quantification of the gene expression level for each gene was performed according to the comparative method 2^-ΔΔCt^, using the averaged ΔCt value on all the LGG samples as tissue control (ΔCt_*ref*_). All 15 loci (represented by 19 probe-sets) were confirmed and validated (Table 4). The qPCR confirmed that all the 15 genes were differentially expressed between infratentorial *versus* supratentorial LGGs in multivariate analysis (RLS) (Table 4). Indeed, the Mann–Whitney test identified 5 out of 15 genes which were also significant in univariate analysis (Table 4). They were: aristaless related homeobox *(ARX),* chemokine (C-X-C motif) ligand 14 *(CXCL14),* G protein-coupled receptor 17 (*GPR17)*, LIM homeobox 2 (*LHX2)* and prostaglandin D2 synthase (*PTGD2S),* whose expressions resulted down-regulated in infratentorial tumours and up-regulated in supratentorial ones. For each sample we measured the qPCR expressions of the 5 selected genes and we verified that the expressions were up- and down- regulated according to the lesion site (Figure 3a).

**Table 4:** qPCR expression values for the selected 15 genes

**Gene name**	**Supratentorial**		**Infratentorial**		**ΔCt median**	**I. *vs. *S.**
	**ΔCt**	**2**^**-ΔΔCt**^	**ΔCt**	**2**^**-ΔΔCt**^		**p-value**
ABBA1	2.19	1.46	2.88	0.9	2.72	-
APOD	-1.33	0.69	-2.46	1.6	-1.87	-
**ARX**	**6.03**	**2.46**	**8.25**	**0.53**	**7.33**	**0.03**
**CXCL14**	**0.84**	**12.61**	**7.17**	**0.18**	**4.5**	**0.009**
FOSB	4.15	0.98	3.84	1.27	4.11	-
FOXG1	4.65	0.7	3.38	1.69	4.14	-
**GPR17**	**2.85**	**1.52**	**8.18**	**0.04**	**3.43**	**0.0049**
**LHX2**	**4.6**	**7.59**	**9.04**	**0.35**	**7.52**	**0.01**
NRXN2	6.28	1.18	6.52	0.99	6.51	-
**PTGD2S**	**1.34**	**1.66**	**5.4**	**0.1**	**2.08**	**0.0049**
SDC3	2.9	0.94	1.79	2.03	2.81	-
SNX22	6.98	0.73	6.34	1.11	6.5	-
SPOCK1	3.99	0.88	3.06	1.69	3.81	-
TIMP4	2.62	0.92	2.42	1.05	2.48	-
ZFHX4	-0.43	0.85	-0.84	1	-0.84	-

qPCR values for dataset 1 (34 samples) and 2 (18 samples). Gene expressions were significantly different for all listed genes by multivariate analysis (RLS). The genes in bold are those passing the Mann–Whitney test (only its significant p-values are reported on the right column). S: Supratentorial, I: Infratentorial.

Next, to build a multivariate statistical model on the qPCR data and validate the results of the microarray analyses we applied RLS classification method. The LOO-cross-validation error was 25%. The multivariate model obtained with the RLS analysis was used to classify the available samples, achieving an accuracy of 91%. The classification results were compared to those achieved by the multivariate model from the l_1_l_2_ analysis on the microarray data (Figure 3b). Needless to say, we could only compare the results on the samples in dataset 1 measured both with microarray and qPCR (n = 34). 29 out of 34 (85%) were correctly classified by both methods. The microarray and qPCR analyses could not correctly classify 3 cases, two of which were assigned to the right class by the qPCR model. One sample was incorrectly classified by both approaches hence 33 out of 34 were associated to the right class by either method.

### Microarray-based differences of infratentorial *versus* supratentorial PAs

Similarly, we conducted the analysis only on 27 PAs out of 40 LGGs (the FA was excluded), whose 17 arising in infratentorial and 10 in supratentorial regions, see Table 1. Thank to the application of the l_1_l_2_ selection framework to the dataset, a list of 136 highly discriminative probe-sets corresponding to 82 genes was selected (see Additional file 5). The system performance was evaluated by its corresponding cross-validation error, as low as 15.4%.

**Figure 4:**
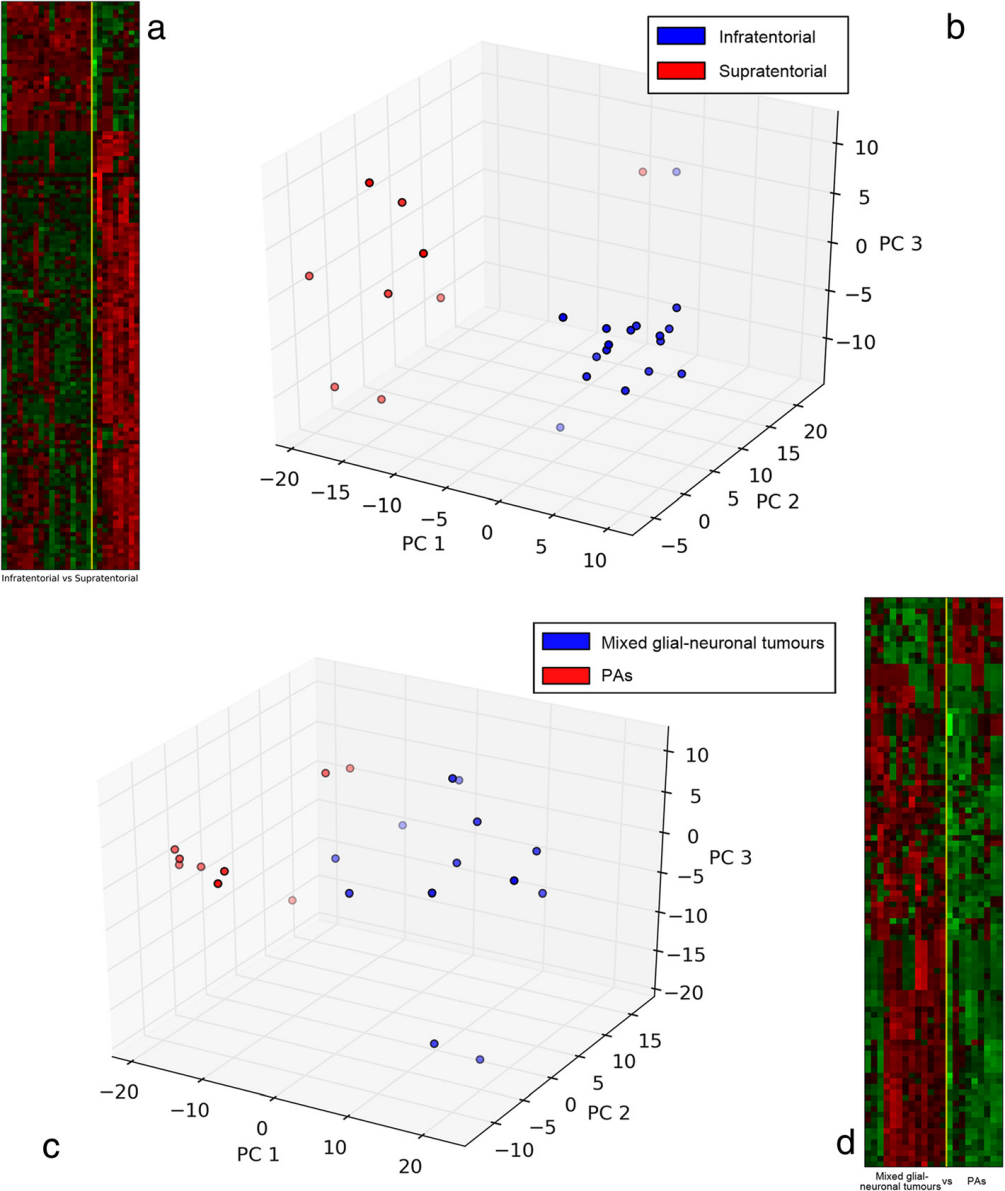
**Molecular fingerprinting sub-classify infratentorial from supratentorial PAs as well as separate mixed glial-neuronal tumours from PAs. a)** heatmap - infratentorial *vs.* supratentorial PAs. Heatmap plot of the gene expression submatrix of the 27 PAs restricted to the 136 probesets selected by the l_1_l_2_ feature selection. The tumours are grouped according to the lesion site (infratentorial *vs.* supratentorial). Each column represents a sample and each row is associated to a probe-set. The relative expression of the probe-sets is normalized ranging from 0 (blue, under-represented) to 1 (red, over-expressed), **b)** This figure illustrates a 3-dimensional visualization of the dataset restricted to the 136 selected probe-sets. The 3D representation is obtained by projecting the data submatrix onto its 3 principal components *i.e.* the components of maximum variance. Red circles represent the supratentorial and the blue circles the infratentorial PAs; **c)** This figure illustrates a 3D projection of the dataset restricted to the 103 selected probe-sets for the supratentorial tumours: mixed glial-neuronal tumours *vs*. PAs. The 3D representation is obtained by projecting the data submatrix onto its 3 principal components *i.e*. the components of maximum variance. Red circles represent the PAs and the blue circles the mixed glial-neuronal tumours; **d)** Heatmap plot of the gene expression submatrix of the 22 supratentorial tumours restricted to the 103 probe-sets selected by the l_1_l_2_ feature selection. The tumours are grouped according to the histotype (mixed glial-neuronal tumours *vs.* PAs) Each column represents a sample and each row is associated to a probe-set. The relative expression of the probe-sets is normalized ranging from 0 (blue, under-represented) to 1 (red, over-expressed).

The strong discriminative power of the 136 selected probe-sets is visualized in Figure 4a,b. As shown in Figure 4b, the two classes of PA related to site of lesion are clearly separated in the multidimensional space. Again, the functional characterization of the gene signature performed with different web-tools, shows distinct processes enriched, as following: nervous system development, cell morphogenesis and cell adhesion, MAPK cascade, and chemotaxis. Moreover, the main pathways coming out are: chemokine signaling, transforming growth factor beta (TGF-β) signaling, MAPK signaling, Glioma, and WNT signaling pathways. The gene signature of this question is almost completely included in the larger LGG gene signature (62 common genes), but nineteen genes were specifically related to PA histotype as a group (see genes reported in bold in Additional file 5). Intriguingly, gene ontology analysis showed that distinct genes among the 19 related to PA, create a network within the TGF-β signaling pathway [KEGG ID: 04350].

### Microarray-based differences of supratentorial tumours: mixed glial-neuronal tumours *versus* PAs

Finally, the same analysis pipeline was applied to 22 supratentorial LGGs to distinguish mixed glial-neuronal tumours (12 samples) from PAs (10 samples), see Table 1. The l_1_l_2_ algorithm selected a list of 103 highly discriminative probe-sets corresponding to 70 genes as shown in Additional file 6. Even in this case, the system performance with its corresponding cross-validation error, *i.e*., 27% was analyzed.

The list of 70 genes, able to discriminate mixed glial-neuronal tumours *vs.* PAs (Figure 4c,d), includes genes involved in the extracellular matrix organization, forebrain development, and neuron differentiation such as distal-less homeobox 1 and 2 (*DLX1, DLX2*), immune response, such as hemoglobin alpha 1 and 2 (*HBA1/2),* chemokine (C-X-C motif) ligand 12 *(CXCL12*), chemokine (C-C motif) ligand 5 *(CCL5*), and metabolic proteins (Additional file 4). Distinct pathways are enriched: toll-like receptor signaling, focal adhesion, extracellular matrix constituents and remodeling machinery, and extracellular matrix (ECM) receptor interaction pathways. Interestingly, the presence of gene family of collagen such as collagen type I, alpha 1 and 2 (*COL1A1, COL1A2),* collagen type III, alpha1 *(COL3A1),* collagen type V, alpha 1 *(COL5A1*), and collagen type VI, alpha 2 and 3 *(COL6A2*, *COL6A3*) are significantly represented in mixed glial-neuronal tumours.

## Discussion

A major emphasis has historically been placed on stratifying LGGs diagnosis or therapy on the basis of pathological and molecular genetic criteria. However, the increasing application of molecular approaches is transforming the way to categorize these tumours, since it seems that histologically comparable lesions may exhibit diverse patterns of gene expression and genomic alterations [5-7,9,19,20]. This investigation has focused on the identification of a specific gene signature based on high-throughput techniques that provide a genome-wide snapshot of LGGs with respect to both distinct lesion site in the brain and histotype.

Although an abundance of data is available on gene expression profiles of LGGs, they are often conflicting. Indeed, statistical methods for evaluation and interpretation of microarray data are still evolving. We successfully adopted an analysis workflow (Figure 1) able to overcome a major criticality in high-throughput studies, that is to find robust, reproducible and biologically sound results [16,46]. Details of the workflow description are reported in Additional file 1.

### Brain region-specific gene signature among LGGs

Question (1) was used to assess the procedure and represent the first example of biologically validated l_1_l_2_ framework with an independent methodology. Indeed, this query is the one with more samples available as well as the one already investigated in previous works [9,19]. The provided outcome from l_1_l_2_ was a list of 331 probe sets (Additional file 3), corresponding to 206 loci, above 70% of frequency. l_1_l_2_ produces a multi-gene model and only a multidimensional representation can correctly visualize its strong discriminative power (Figure 2b). The figure shows that the infratentorial tumours group is spatially separated from the supratentorial counterpart.

Our analysis identified various interesting genes which encode cell adhesion molecules, ECM, extracellular matrix, lipid metabolism, CNS development, cell differentiation, transcription regulation, and invasion-related proteins. Unlike Potter et al. reported [21], our results are in line with previous findings that clearly defined the existence of PA subgroups. Indeed, 14 out of 206 differentially expressed genes (reported in bold in Table 2) were reported by previous studies [9,19,20]. Wong and colleagues identified two subgroups of PA reporting a list of significant differentially expressed genes involved in cell adhesion, regulation of cell growth, cell motility, and angiogenesis [19]. Sharma and colleagues reported differential expression of genes playing a role in forebrain development as *LHX2* and nuclear receptor subfamily 2, group E, member 1 *(NR2E1),* and hindbrain development as paired box gene 3 (*PAX3)* and iroquois homeobox protein 2 *(IRX2),* able to stratify infratentorial from supratentorial PAs [9]. The comparison with the Sharma’s data, the only comparable, inasmuch as homologous for case selection, sample processing and Affymetrix platform, allowed us, even using our own statistical approach, to identify five genes (*LHX2, NR2E1, PAX3, IRX2,* and zinc finger homeobox 4, *(ZFHX4)* common to both analyses.

To investigate paediatric LGG development related to site of lesion (infratentorial *vs*. supratentorial) [47], we next proceeded by selecting those candidate genes that were most represented among all the high-ranked pathways for the validation process by using our in-house designed qPCR systems on 52 samples (34 samples belonged to dataset 1, while 18 samples were from dataset 2). Finally, the list of candidates comprised 19 probe-sets corresponding to 15 loci in total (Table 3). We validated the generalization ability of the 15 gene signature by applying a multivariate statistical model on the qPCR data of dataset 1 (34 samples). Such multivariate model, obtained with a RLS analysis, was used to assign the samples to a group and the classification results were compared to the l_1_l_2_ microarray-based model (Figure 3b). The two independent methods have good performances, being able to associate 33 out of 34 samples to the right class. Moreover, 5 out of 15 genes emerged from the univariate Mann–Whitney test on the qPCR data, confirming and enhancing the LGG differences in infratentorial as compared with supratentorial regions, see Table 4 and Figure 3a. As shown in Figure 3a, a group of 4 genes (*ARX, GPR17, LHX2* and *CXCL14*) well stratified LGGs between infratentorial and supratentorial tumours. *ARX* is a homeobox-containing gene expressed during development. This gene is involved in CNS development and in cell proliferation in forebrain [GO:0021846]. Mutations in this gene cause X-linked mental retardation and epilepsy. To the best of our knowledge, *ARX* was never associated with LGGs. *GPR17* is a G-protein involved in signal transduction [GO:0007165]. *LHX2* is downregulated in infratentorial tumours as already reported [9]. *CXCL14* is a chemokine associated with tumour development [GO:0006995], and *PTDG2S* whose functions are associated to lipid metabolism [GO:0006633], might be involved in controlling the proliferation rate of LGGs.

Additionally, the predominant terms related to pathways consisted of MAPK signaling pathway, containing at least 12 genes, followed by chemokine signaling pathway with 8 genes enriched. These findings reinforce the observations of several consecutive articles about aberrant activation of the mitogen-activated protein kinase (MAPK) pathway in LGGs [48]. The identification of a brain region-specific gene signature suggests that LGGs at different sites may be distinct in terms of biological properties and tumorigenesis despite the same histology. *KIAA1549:BRAF* fusions were analyzed in the LGG cohort and we found the gene fusion slightly more frequent in infratentorial (38.5%) *versus* supratentorial (25%) tumours, while we didn’t note any difference for *BRAF* V600E mutation. Moreover, we did not identify significantly improved progression-free survival in tumours with gene-fusions or *BRAF* V600E mutation.

### Identification of a subgroup of 19 genes specifically related with PA histotype

Next, to molecularly characterize PA able to distinguish infratentorial *versus* supratentorial, l_1_l_2_ analysis were conducted only on 27 PAs out of 37 LGGs, whose 17 arising in infratentorial and 10 in supratentorial regions, see Table 1. A gene signature of 82 genes (see Additional file 5) well distinguishes PA arising supratentorial *versus* infratentorial regions (Figures 4a,b). Significant biological processes represented include GO terms of nervous system development, cell morphogenesis, cell differentiation and cell adhesion, MAPKKK cascade, chemotaxis, and regulation of neurogenesis. We found that, together with *ARX,* forkhead box G1 *(FOXG1)* was strongly represented in PA. *FOXG1* is an oncogenic transformer which could play an important role in controlling both cell proliferation and forebrain cell differentiation in PA [21,49-51].

Through the comparison of gene lists between LGG and PA, we found 19 genes specifically related with PA histotype as a group (genes in bold in Additional file 5). The functional analysis showed that several genes create a network within the (TGF-β)-signaling pathway. This pathway possess a dual role in oncogenesis. In some tumour types, *i.e*., in high-grade gliomas, TGF-beta becomes an oncogenic factor, while it is also considered a tumour suppressor factor in normal epithelial cells and astrocytes. Moreover, noncanonical TGF-beta signaling pathways interact, through RSmads molecules, with MAPK signaling pathway [52]. Thanks to this interaction, it is likely to assume an active involvement of TGF-beta signaling pathway in the PA development.

Our analysis shows a strong difference between supratentorial and infratentorial PAs. In fact, cerebellar PAs, corresponding to the classical description of the biphasic tumour with compact areas with piloid cells and Rosenthal fibers and microcistic areas with granular eosinophilic bodies [3], seem to be defined by a specific gene signature *versus* supratentorial PAs. Therefore, this molecular fingerprint is able to better sub-classify such a morphologically heterogeneous tumours.

### Neurogenesis, cell motility and cell growth genes dichotomize mixed glial-neuronal tumours *versus* PAs

Finally, the analysis on 22 supratentorial LGGs identified a list of 70 genes (see Additional file 6) able to dichotomize mixed glial-neuronal tumours *versus* PAs (Figure 4c,d). The signature consists of genes encoding adhesion, ECM-receptor interaction, matrix extracellular organization, neurogenesis, immune response, and metabolic proteins. Several genes are components of collagen gene family whose functions are associated with extracellular matrix (ECM) reorganization. Intriguingly, changes in expression of genes controlling neurogenesis (*DLX1, DLX2*), cell growth such as insulin-like growth factor 2 (*IGF2),* insulin-like growth factor binding protein 6 *(IGFBP6)* and latent transforming growth factor beta binding protein (*LTBP2*), cell motility such as l1 cell adhesion molecule (*L1CAM), COL3A1* and integrin, alpha 8 (*ITGA8*), and interactions with the surrounding environment such as lumican (*LUM), COL1A1, COL6A3* and periostin, osteoblast specific factor *(POSTN*) appear to be linked to the presence of neuronal cell component.

Because of their rare occurrence, little is yet known about the molecular pathology of mixed glial-neuronal neoplasms and the cytogenetic and molecular genetic studies reported are very few [53-55]. Our findings show the complexity and vitality of these tumours, shedding some light on features such their richness in connective tissue and, they point to some interesting candidate genes (*i.e., DLX1, DLX2*) worth further investigations that could help the pathologists in the differential diagnosis.

From a biological point of view, it is remarkable that the mixed glial-neuronal tumours are strikingly separated from PAs, allowing us to look differently at mixed glial-neuronal tumours in which, generally, the glial component catches the attention of the pathologists and contributes to grading. Our findings, indeed, shed some light on the biological complexity of the mixed glial-neuronal tumours, still poorly known. It remains to be established if mixed glial-neuronal tumours differ from PAs because of their ganglion-like component or because of their glial one or both. What seems indubitable is that the ganglion cell component is not a bystander. Future functional studies are needed to evaluate these targets in paediatric mixed glial-neuronal tumours *versus* PAs but evidence supports a role for these gene candidates in tumorigenesis.

## Conclusion

The identification of a brain region-specific gene signature suggested that LGGs at different sites may be distinct in terms of biological properties and tumorigenesis. The success of our methodology carries implications for improving the diagnosis and possibly prognosis of LGGs. The method efficiently finds and ranks genes that can distinguish one histotype from another. In addition, we performed clustering and classification of GO categories and possibly altered pathways on the basis of gene expression in infratentorial *versus* supratentorial LGGs, in particular in the PAs, and among supratentorial tumours, in mixed glial-neuronal tumours *versus* PAs.

The analyses reinforce previous observations about aberrant activation of the mitogen-activated protein kinase (MAPK) pathway in LGGs but, still point to an active involvement of TGF-β signaling pathway in the PA development and, emphasize some interesting candidate genes worth further investigations for the mixed glial-neuronal tumours. Considering the high clinical and biological relevance of the disease, as these tumours are detrimental to children, and since the genetic background of paediatric glial tumours is still unsatisfied, this methodological work could mark the starting line. A genotype-phenotype correlation of LGGs is instrumental to improve classification and differential diagnosis. Impact of molecular classification will likely change how LGGs are both diagnosed and treated henceforward. This paper thus provides a novel global view of the molecular differences between infratentorial and supratentorial LGGs.

Further investigation and validation by experiments should be targeted to the exploration of a deeper genotype-phenotype correlation in those LGG cases who undergo malignant transformation.

## Abbreviations

PA: Pilocytic astrocytoma; WHO: World Health Organization; LGG: Low Grade Glioma; DIA: Desmoplastic infantile astrocytoma; DIG: Desmoplastic infantile ganglioglioma; GG: Ganglioglioma; FA: Diffuse fibrillary astrocytoma; CNS: Central nervous system; NF1: Neurofibromatosis type 1; RLS: Regularized least squares; DAVID: Database for annotation, visualization and integrated discovery; KEGG: Kyoto encyclopedia of genes and genomes; GO: Gene ontology; BP: Biological process; GenMAPP: Gene map annotator and pathway profiler; EASE: Expression analysis systematic explorer; EGAN: Exploratory gene association network; MAPK: Mitogen-activated protein kinase; TGF-β: Transforming growth factor-beta; CAM: Cell adhesion molecule; ECM: Extracellular matrix; ARX: Aristaless related homeobox; CXCL14: Chemokine (C-X-C motif) ligand 14; GPR17: G protein-coupled receptor 17; LHX2: LIM homeobox 2; NR2E1: Nuclear receptor subfamily 2, group E, member 1; PAX3: Paired box gene 3; IRX2: Iroquois homeobox protein 2; PTGD2S: Prostaglandin D2 synthase; ACTB: Beta actin; PKM2: Pyruvate kinase; B2M: Beta-2-microglobulin; DLX1/2: Distal-less homeobox 1, 2; HBA1/2: Hemoglobin alpha 1, 2; CXCL12: Chemokine (C-X-C motif) ligand 12; CCL5: Chemokine (C-C motif) ligand 5; COL1A1/2: Collagen type I, alpha 1, 2; COL3A1: Collagen type III, alpha1; COL5A1: Collagen type V, alpha 1; COL6A2/3: Collagen type VI, alpha 2, 3; FOXG1: Forkhead box G1; IGF2: Insulin-like growth factor 2; IGFBP6: Insulin-like growth factor binding protein 6; LTBP2: Latent transforming growth factor beta binding protein; L1CAM: L1 cell adhesion molecule; ITGA8: Integrin, alpha 8; LUM: Lumican; POSTN: Periostin, osteoblast specific factor; TIM4: TIMP metallopeptidase inhibitor 4; APOD: Apolipoprotein D; ZFHX4: Zinc finger homeobox 4; SDC3: Syndecan 3; NRXN2: Neurexin 2; SNX22: Sorting nexin 22; ABBA1: Metastasis suppressor 1 like; SPOCK1: Testican 1; FOSB: FBJ murine osteosarcoma viral oncogene homolog B; KIAA1549: KIAA1549; BRAF: v-raf murine sarcoma viral oncogene homolog B1.

## Competing interests

The authors declare that they have no competing interests.

## Authors’ contributions

SM and AR performed the experiments, interpreted the data and wrote the manuscript. AB performed statistical data analysis, participated in guiding some experiments and wrote the manuscript. SM, CM, DF, GW and KN participated in performing statistical data analysis. PN carried out pathological data review, contributed to the study design and with RB, provided research and editorial assistance. MH and SP provided assistance for the clinical data. GM provided neuroradiological data. AC, GP and provided tissue samples from the surgery. MLG provided tissue samples from the tissue-bank and clinical data, and with VC contributed to the study design, collaborated to the research and review the manuscript. AV contributed to the statistical analyses design and review the manuscript. All authors read and approved the final manuscript.

## Pre-publication history

The pre-publication history for this paper can be accessed here:

http://www.biomedcentral.com/1471-2407/13/387/prepub

## Supplementary Material

Additional file 1 **Workflow for high-throughput gene-expression studies.** Description and discussion, including references, of the pipeline used for gene-expression studies. Click here for file

Additional file 2 **qPCR systems.** Primers sequences and the amplification conditions of the 15 selected genes validated by qPCR, including 3 genes used as the endogenous control for each tumour specimen. Click here for file

Additional file 3 **Selected probe-sets by l**_**1**_**l**_**2 **_**for LGG related to the site of lesion.** List of 331 probe-sets selected by the l_1_l_2_ procedure for LGG related to the site of lesion (infratentorial *versus* supratentorial). For each probe-set we report the corresponding Gene ID and its frequency score (f.s.). Click here for file

Additional file 4 **Microarray-based differences of LGG related to site of lesion.** A brief comment of each locus, listing the main protein functions and including references, for the 15 selected genes that significantly discriminate infratentorial *versus* supratentorial LGGs. Click here for file

Additional file 5 **Pilocytic Astrocytomas - infratentorial *vs *supratentorial, selected probe-sets by the *l***_***1***_***l***_***2 ***_**for PA related to the site of lesion.** List of 136 probe-sets selected by l_1_l_2_ for infratentorial *vs* supratentorial PAs. For each probe-set we report the Gene ID and its frequency score (f.s.). Genes reported in bold are those specific to PA histotype as a group. Click here for file

Additional file 6 **Supratentorial tumours:** mixed glial-neuronal tumours *vs.* PAs, selected probe-sets by the *l*_*1*_*l*_*2.*_ List of 103 probe-sets selected by the l_1_l_2_ procedure. For each probe-set we report the corresponding Gene ID and its frequency score (f.s.). Click here for file
