# Supplementary material for: Molecular fingerprinting reflects different histotypes and brain region in low grade gliomas
Source: BMC Cancer. 2013 Aug 15;13:387. doi: 10.1186/1471-2407-13-387 (PMC3765921; doi:10.1186/1471-2407-13-387)
Supplement: Additional file 1 — Workflow for high-throughput gene-expression studies. Description and discussion, including references, of the pipeline used for gene-expression studies. [file 1471-2407-13-387-S1.pdf]

Additional file 1: Workflow for high-throughput gene-expression studies

The pipeline is a supervised machine learning workflow consisting in 3 main consecutive phases:

Initially (Phase 1), the case selection was carefully carried out to avoid biased and irreproducible results. Moreover, tumour processing was particularly challenging because in paediatric patients the amount of tissue might be very small.

In Phase 2, the main aim of our statistical analysis is to select those variables (probe sets) that allow for an optimal classification according to both histotype and brain site of origin. This problem is known as binary classification problem. A classifier is a rule assigning a patient to one of two classes and its quality is measured by the expected misclassification error on old as well as new patients. In our case the aim is not only to build a classifier but also to verify which are the most discriminative probe sets. Though such a problem is well known in statistics and algorithms are available, the application to microarray data analysis poses non trivial problems, the main reason being the large number of variables (genes) compared to the small number of examples (patients). In this kind of regime avoiding overfitting becomes crucial and statistical methods to assess the reliability of the obtained results are needed. Note that our goal is to have some confidence on the classification performance of the obtained classifier and the stability of the obtained list. To this end, we apply a machine learning method based on regularization ($\ell_1\ell_2$) to select the candidate probe-sets. $\ell_1\ell_2$ is multivariate, correlation aware and sparsity inducing and it is cast in a selection-bias free framework that also provides a good prediction performance [1,2] and a stability score based on frequency. In general, the number of genes produced by the analysis should be sufficiently small to be potentially useful for clinical diagnosis/prognosis or as candidates for functional analysis to determine whether they could serve as useful targets for therapy. In order to reduce the probe-set list at a reasonably acceptable length to be successively validated, we select a subset of genes following a functionally based criterion based on the information provided by all the databases in use. This enables us to distinguish those genes mostly represented in the majority of the relevant pathways.

The Phase 3 consists of a validation process to confirm the results and verify their generalization ability. We validate in silico as well as on the biological level, by means of independent techniques [3], providing experimental verification of gene-expression signatures [4,5]. The genes are indeed analyzed by means of qPCR, the gold-standard for the validation of microarray based studies [6-8], because it measures the same mRNA variables that were measured by the microarray, as opposed to other techniques (i.e. Western blotting) that investigate the protein level. This allows us to avoid measurement bias related to post-translational modification that might influence the proteome expression but not the transcriptome.

References

1. De Mol C, Mosci S, Traskine M, Verri A: **A regularized method for selecting nested groups of relevant genes from microarray data.** *Journal of Computational Biology* 2009, **16**:1-15.

2. Barla A, Mosci S, Rosasco L, Verri A: **A method for robust variable selection with significance assessment**. *Proceedings of ESANN* 2008 .

3. Canales RD, Luo Y, Willey JC, Austermiller B, Barbacioru CC, et al: **Evaluation of dna microarray results with quantitative gene expression platforms.** *Nature biotechnology* 2006, **24**:1115-22.

4. Yuen T, Wurmbach E, Pfeffer RL, Ebersole BJ, Sealfon SC: **Accuracy and calibration of commercial oligonucleotide and custom cdna microarrays**. *Nucleic Acids Res* 2002, **30**:e48.

5. Kothapalli R, Yoder SJ, Mane S, Loughran TP: **Microarray results: how accurate are they?.** *BMC Bioinformatics* 2002, **3**:22.

6. VanGuilder HD, Vrana KE, Freeman WM: **Twenty-five years of quantitative pcr for gene expression analysis**. *BioTechniques* 2008, **44**:619-26.

7. Gyorffy B, Molnar B, Lage H, Szallasi Z, Eklund AC: **Evaluation of microarray preprocessing algorithms based on concordance with rt-pcr in clinical samples**. *PLoS ONE* 2009, **4**:e5645.

8. Bustin SA: **Developments in real-time pcr research and molecular diagnostics.** *Expert Rev Mol Diagn* 2010, **10**:713-5.
