# Supplementary material for: Molecular fingerprinting reflects different histotypes and brain region in low grade gliomas
Source: BMC Cancer. 2013 Aug 15;13:387. doi: 10.1186/1471-2407-13-387 (PMC3765921; doi:10.1186/1471-2407-13-387)
Supplement: Additional file 4 — Microarray-based differences of LGG related to site of lesion. A brief comment of each locus, listing the main protein functions and including references, for the 15 selected genes that significantly discriminate infratentorial versus supratentorial LGGs. [file 1471-2407-13-387-S4.doc]

**Additional file 4: Microarray-based differences of LGGs related to site of lesion**

We here briefly comment each locus, listing the main protein functions (source: [1]).

FOXG1 belongs to the forkhead family of transcription factors. Its specific function has not yet been determined; however, it may play a role in the development of telencephalon.

GPR17 is a G-protein-coupled receptor involved in the signal transduction and its isoform hGPR17-S is abundantly expressed in the brain.

CXCL14 belongs to the cytokine gene family which encodes secreted proteins involved in immunoregulatory and infiammatory processes.

ARX is a homeobox-containing gene expressed during the Central Nervous System (CNS) development.

LHX2 encodes a highly conserved transcription factor characterized by the LIM domain, it regulates the formation of the cortical hemispheres [2] and it is also involved in the tumorigenesis [3].

The proteins encoded by the TIMP4 family are inhibitors of the matrix metalloproteinases, a group of peptidases involved in degradation of the extracellular matrix.

APOD encodes a glycoprotein involved in various cellular processes. It is highly expressed in neuronal structures and tissues active in steroid hormone metabolism and it is already known to be a potential universal target for glial tumours therapy [4].

PTGD2S encodes a a typical brain enzyme that catalyzes the conversion of prostaglandin H2 (PGH2) to the neuro-modulator postaglandin D2 (PGD2).

ZFHX4 homeodomain-zinc finger protein is expressed in neuronal differentiation manner.

SDC3 encodes a protein of the syndecan proteoglycan family. It may play a role in the invasion mechanism of glioblastomas [5].

NRXN2 encodes a member of the neurexin gene family. The products of the neurexin genes function as cell adhesion molecules and receptors in the vertebrate nervous system.

SNX22: the sorting nexins (SNXs) constitute a large group of PX domain-containing proteins that play critical roles in protein trafficking.

SPOCK1 encodes the protein core of a seminal plasma proteoglycan containing chondroitin- and heparan-sulfate chains. The protein's function is unknown, although similarity to thyropin-type cysteine protease-inhibitors suggests its function may be related to protease inhibition.

ABBA-1 belongs to the MTSS1 family and may be related to cancer progression or tumour metastasis in a variety of organ sites.

FOSB encodes a leucine zipper protein, being implicated as regulator of cell proliferation, differentiation, and transformation.

**References**

1. Huang DW, Sherman BT, Lempicki RA: **Systematic and integrative analysis of large gene lists using david bioinformatics resources.** *Nat Protoc* 2009, **4**:44-57.

2. Bulchand S, Grove EA, Porter FD, Tole S: **Lim-homeodomain gene lhx2 regulates the formation of the cortical hem**. *Mech Dev* 2001, **100**:165-75.

3. Bach I: **The lim domain: regulation by association**. *Mech Dev* 2000, **91**:5-17.

4. MacDonald TJ, Pollack IF, Okada H, Bhattacharya S, Lyons-Weiler J: **Progression-associated genes in astrocytoma identified by novel microarray gene expression data reanalysis**. *Methods Mol Biol* 2007, **377**:203-22.

5. Watanabe A, Mabuchi T, Satoh E, Furuya K, Zhang L, et al: **Expression of syndecans, a heparan sulfate proteoglycan, in malignant gliomas: participation of nuclear factor-kappab in upregulation of syndecan-1 expression**. *J Neurooncol* 2006, **77**:25-32.
